# Hydrocephalus with panventricular enlargement as the primary manifestation of neurosarcoidosis: a case report

**DOI:** 10.1186/1752-1947-7-240

**Published:** 2013-10-14

**Authors:** Sadaharu Tabuchi, Tetsuji Uno

**Affiliations:** 1 Department of Neurosurgery, Institute of Neurological Sciences, Tottori University School of Medicine, 36-1 Nishi-cho, Yonago 683-8504, Japan; 2 Department of Neurosurgery, Tottori Prefectural Central Hospital, 730 Ezu, Tottori 680-0901, Japan

**Keywords:** Hydrocephalus, Magnetic resonance imaging, Neurosarcoidosis, Shunt

## Abstract

**Introduction:**

Hydrocephalus is very uncommon in neurosarcoidosis. To date, there have been only five reported cases of hydrocephalus occurring as the first manifestation of neurosarcoidosis. Such a presentation in a previously healthy patient is challenging to diagnose.

**Case presentation:**

A 31-year-old Japanese man who had no relevant past history other than sinusitis was admitted to our institution complaining of low-grade fever and mild headache. He was alert and neurologically intact. No respiratory symptoms were observed. Laboratory examination revealed mild elevation of erythrocyte sedimentation rate and serum CD4/CD8 ratio. Serum angiotensin-converting enzyme level was in the normal range. His cerebrospinal fluid showed mild pleocytosis and increased protein level. A chest X-ray revealed bihilar lymphadenopathy with normal lung parenchyma. Computed tomography of his head showed remarkable hydrocephalus with dilatation of all ventricles, particularly the fourth. Gadolinium-enhanced magnetic resonance imaging demonstrated leptomeningeal millet seed-like enhancement and multiple small enhancing lesions along the Virchow–Robin spaces. These findings strongly suggested a chronic inflammatory disease such as neurosarcoidosis. To treat the hydrocephalus, a ventriculoperitoneal shunt was inserted. The postoperative course was satisfactory. After surgery, nasal and skin biopsies were performed and pathological analysis revealed non-caseating granulomas consistent with sarcoidosis. The findings of gallium scintigraphy also supported the diagnosis of sarcoidosis. We obtained the definitive diagnosis of sarcoidosis 3 weeks after admission from the pathological findings by the nasal and skin biopsies, and corticosteroid therapy was started after that.

**Conclusion:**

We present a rare case of neurosarcoidosis manifesting as acute hydrocephalus with dilatation of all ventricles, particularly the fourth. As hydrocephalus due to neurosarcoidosis has high morbidity and mortality, early diagnosis and proper treatment are particularly important.

## Introduction

Sarcoidosis is a multisystem disease of unknown origin, most commonly affecting young adults. It varies in incidence among countries [1]; with high rates in Sweden (64 per 100,000 population) and the United States of America (10.9 per 100,000) compared to Japan (1 per 100,000). Neurological involvement occurs in only 5% of patients with sarcoidosis. A prospective study showed that hydrocephalus occurred in 6% of patients with neurosarcoidosis [2], but most of these patients had previously diagnosed systemic sarcoidosis which required corticosteroid therapy. An atypical presentation of sarcoidosis, such as acute hydrocephalus in a previously healthy patient, is extremely rare and difficult to diagnose. We report the case of a 31-year-old Japanese man who had remarkable hydrocephalus of all ventricles as the initial manifestation of systemic sarcoidosis. He was treated with a ventriculoperitoneal (VP) shunt and corticosteroid therapy.

## Case presentation

**Figure 1:**
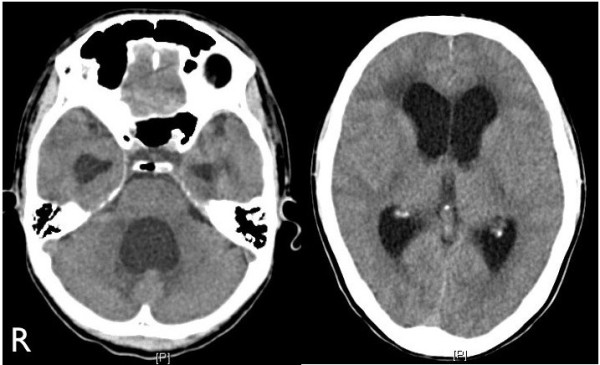
Computed tomography performed at admission showed remarkable hydrocephalus with dilatation of all ventricles, particularly the fourth ventricle.

**Figure 2:**
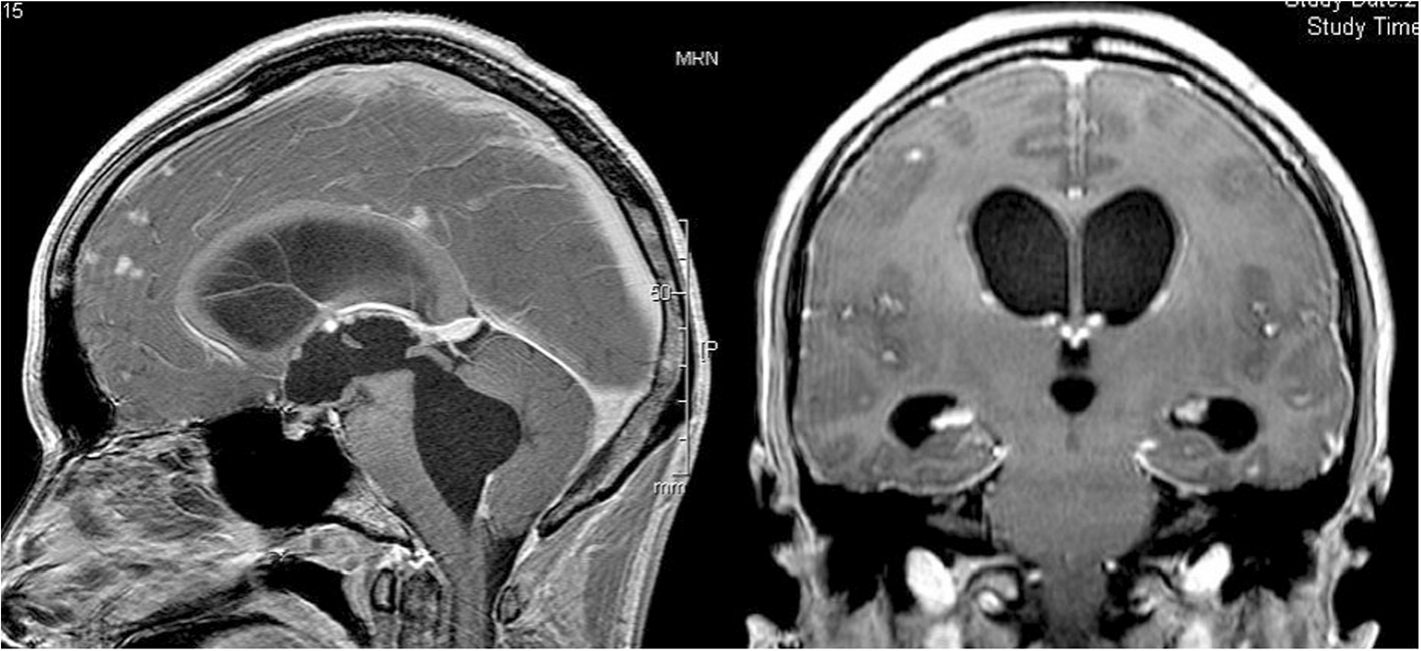
Preoperative contrast-enhanced magnetic resonance imaging showed millet seed-like enhancement along the third ventricular wall, multiple small enhanced lesions along the Virchow–Robin spaces, and abnormal enhancement near the foramen of Magendie.

A 31-year-old Japanese man who had no relevant medical history other than sinusitis was admitted to our institution complaining of low-grade fever and mild headache which had progressively worsened. He was alert, and a neurological examination revealed no focal signs. Fundoscopic examination showed no papilledema or other abnormalities. No respiratory abnormalities were observed. His complete blood count and routine biochemistry were within normal range. Although C-reactive protein was negative, erythrocyte sedimentation rate was mildly elevated at 14mm/hour (normal range: 1 to 9mm/h). His serum level of angiotensin-converting enzyme was 10.7U/I (normal range: 8.3 to 21.4U/I), its cerebrospinal fluid (CSF) level was not tested, his CD4/CD8 ratio was mildly elevated at 3.27 (normal range: 0.9 to 3.1), and tumor markers were negative. Tuberculin reaction was also negative. A chest X-ray showed bihilar lymphadenopathy with normal lung parenchyma. Computed tomography (CT) showed extensive mediastinal, hilar, and paratracheal lymphadenopathy. A CT of his head showed remarkable hydrocephalus with dilatation of all the ventricles, particularly the fourth, but it did not reveal the etiology (Figure 1). Chiari-type I malformation was excluded on magnetic resonance (MR) imaging. Cine-MR imaging showed good patency of the cerebral aqueduct and absence of a CSF flow signal in the area of the cisterna magna. Gadolinium-enhanced T1-weighted imaging showed millet seed-like leptomeningeal enhancement and multiple small enhancing lesions along the Virchow Robin spaces (Figure 2). Abnormal enhancement was also observed around the fourth ventricular outlet. These findings strongly suggested a chronic inflammatory disease such as neurosarcoidosis. The findings of gallium scintigraphy supported a diagnosis of sarcoidosis. In light of the clinical and fundoscopic findings we performed a lumbar puncture. Opening pressure was 14cm water (H_2_O); the CSF was transparent and showed mild pleocytosis (lymphocytosis) and a total protein level of 213mg/dL. CT-cisternography revealed a sparse backflow of contrast medium into the fourth ventricle, suggesting an incomplete block of CSF circulation at the fourth ventricular outlet. A VP shunt was inserted to treat the hydrocephalus. Intracranial CSF pressure was 20cm H_2_O during surgery, and open biopsies of the arachnoid membrane and the surface of the frontal cortex were performed. That region was not enhanced at preoperative MR imaging. Histopathologic analysis revealed a non-specific abnormal scar-like lesion that was not characteristic of sarcoidosis but did not rule it out. Postoperative CT showed normalization of ventricular size. After surgery, nasal and skin biopsies were performed and pathology revealed non-caseating granulomas consistent with sarcoidosis. We obtained the definitive diagnosis of sarcoidosis 3 weeks after admission from the pathological findings by the nasal and skin biopsies, and corticosteroid therapy was started after that. The clinical course was favorable.

## Discussion

Neurologic findings are observed in approximately 5% of patients with sarcoidosis. The most frequent neurological manifestation is neuropathy affecting one or more cranial nerves. Hydrocephalus is a very uncommon finding. To date, there have been five reported cases of hydrocephalus occurring as the first presentation of neurosarcoidosis [3-7]. The unique finding in the present case is that the patient demonstrated gross hydrocephalus of all ventricles as the first feature of sarcoidosis. The differential diagnosis in such cases should include Chiari type-I malformation, chronic inflammatory diseases such as neurosarcoidosis and tuberculosis, carcinomatous meningitis, metastasis, and lymphoma. We strongly suspected neurosarcoidosis from initial MR imaging findings and laboratory data before insertion of the VP shunt. The previous reported cases underwent emergent ventriculostomy due to increased intracranial pressure. Lukin *et al*. reported a patient with hydrocephalus with panventricular enlargement in whom sarcoidosis had been diagnosed 10 years previously [8]. Autopsy findings showed granulomatous involvement of the meninges in both foramina of Luschka. In the present case, according to the clinical findings, imaging findings, and CSF pressure, the outlets of the fourth ventricle were speculated to be incompletely obstructed by meningeal sarcoidosis.

MR has been reported as the preferred imaging technique to evaluate and monitor neurosarcoidosis [9]. Although there have been no controlled trials of medical treatment for neurosarcoidosis, corticosteroid therapy remains the treatment of choice and has proven useful in many cases. Some patients respond rapidly, whereas others may require long-term therapy [10]. Maniker *et al*. reported a case of multiple shunt failures in a patient with hydrocephalus and sarcoidosis [11]. After removal, the shunt was found to be occluded with non-caseating granulomatous material that had infiltrated its lumen. At present, there is no evidence that steroids can reduce the frequency of shunt failure in such situations. MR imaging is a noninvasive means of monitoring a patient’s response to treatment [12], hence repeated MR imaging of the brain may be mandatory in neurosarcoidosis patients, even if a VP shunt is inserted.

## Conclusion

We present a rare case of neurosarcoidosis manifesting as acute hydrocephalus with dilatation of all ventricles, particularly the fourth. As hydrocephalus due to neurosarcoidosis has high morbidity and mortality, early diagnosis and proper treatment are particularly important.

## Consent

Written informed consent was obtained from the patient for publication of this case report and accompanying images. A copy of the written consent is available for review by the Editor-in-Chief of this journal.

## Competing interests

The authors declare that they have no competing interests.

## Authors’ contributions

ST was the major contributor in writing the manuscript. TU reviewed the manuscript. All authors read and approved the final manuscript.
